# Vector configuration screening failure after defibrillation threshold testing: Should we be concerned?

**DOI:** 10.1016/j.hrcr.2023.03.015

**Published:** 2023-03-30

**Authors:** Ekin C. Uzunoglu, Kevin Liu, Pedro Adrover, Prakash G. Suryanarayana, Claude S. Elayi, John N. Catanzaro

**Affiliations:** ∗ University of Florida Health Science Center, Jacksonville, Florida; † CHI Saint Joseph Hospital – Cardiology, Lexington, Kentucky

**Keywords:** Subcutaneous ICD, Defibrillation threshold testing, Vector configuration screening failure, Transvenous ICD, T-wave oversensing

**Key Teaching Points**

• Acute significant repolarization changes post subcutaneous implantable cardioverter-defibrillator (S-ICD) implant after defibrillation threshold test (DFT) are possible and might alter significantly the automated screening tools.
• It is imperative to make sure that the sensing of S-ICD is intact post DFT testing prior to discharge to allow acute repolarization change resolution, even when the patient passed the screening in the first place.
• The optimal number of passed screening vectors must be taken into account before and after S-ICD implant, especially in patients who only “screen in” with 1 vector configuration.

## Introduction

The subcutaneous implantable cardioverter-defibrillator (S-ICD) is safe and effective in reducing the risk of sudden cardiac death and all-cause mortality among primary prevention patients with a reduced left ventricular ejection fraction.^1^ The S-ICD is noninferior to the transvenous ICD with regard to device-related complications and inappropriate shocks in patients with an indication for an ICD without pacing requirements, and reportedly lower in some cases when contemporary programming filters and discrimination algorithms are used.^1^^,^^2^ However, the S-ICD requires preimplant screening to ensure optimized sensing, which now can be completed with either an ICD programmer or a 12-lead electrocardiogram (ECG). All S-ICD candidates must have at least 1 suitable vector during preimplantation ECG screening to be eligible.^3^

Screening of S-ICD candidates evolved from manual screening tools to automated screening tools (AST) with continuous improvements to the AST to reduce subjectivity while increasing the efficiency and usability.^4^ AST performs screening by first collecting the surface ECG with a surface equivalent of the subcutaneous sensing vectors and then evaluating the QRS complexes for each patient posture tested. After establishing an ECG connection, and verifying a clean ECG, an ECG screening test is run on the Screening tab for both supine and sitting/standing patient postures. AST summarizes the screening results and instructs users to check QRS complex morphology across postures to identify acceptable leads. A physician must confirm the automated results (QRS morphology, stability of sensed QRS complexes, etc) with at least 1 suitable vector for eligibility.^3^^,^^4^ Here, we present the first clinical case of transient S-ICD sensing failure in all vector configurations both in the supine and sitting positions, after high-voltage defibrillation.

## Case report

**Figure 1:**
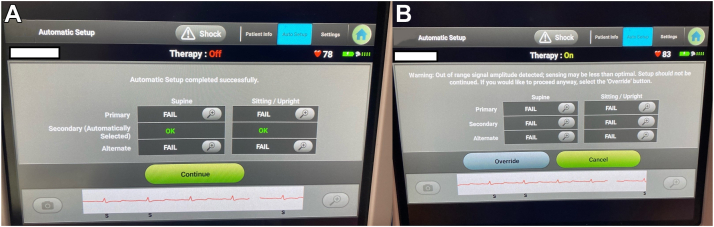
**a:** Subcutaneous implantable cardioverter-defibrillator (S-ICD) interrogator demonstrating a passing secondary vector on the automated screening tool (AST). **b:** S-ICD interrogator demonstrating failure of the secondary vector in supine and sitting positions.

**Figure 2:**
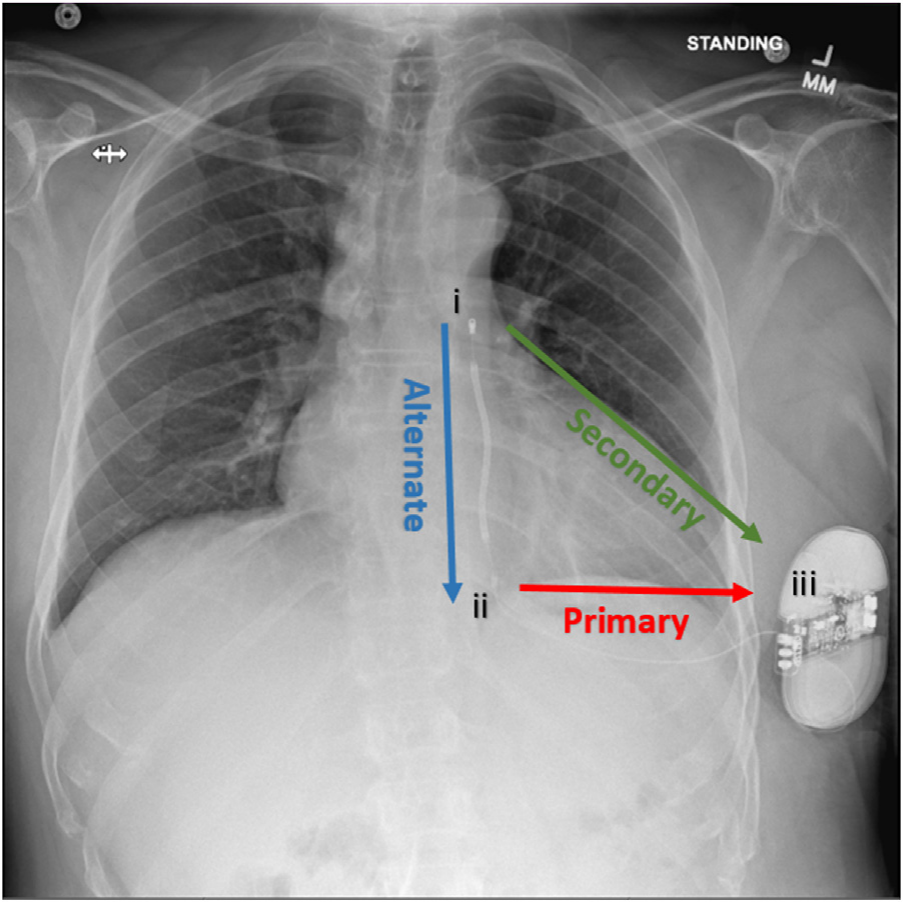
Anteroposterior chest radiograph with subcutaneous implantable cardioverter-defibrillator position and sensing vectors. The 3 available sensing vectors are shown: Primary vector is from proximal electrode ring to can; Secondary vector is from distal electrode ring to can; and Alternate vector is from distal to proximal electrode rings (i represents the distal electrode ring, ii represents the proximal electrode ring, iii represents the pulse generator).

**Figure 3:**
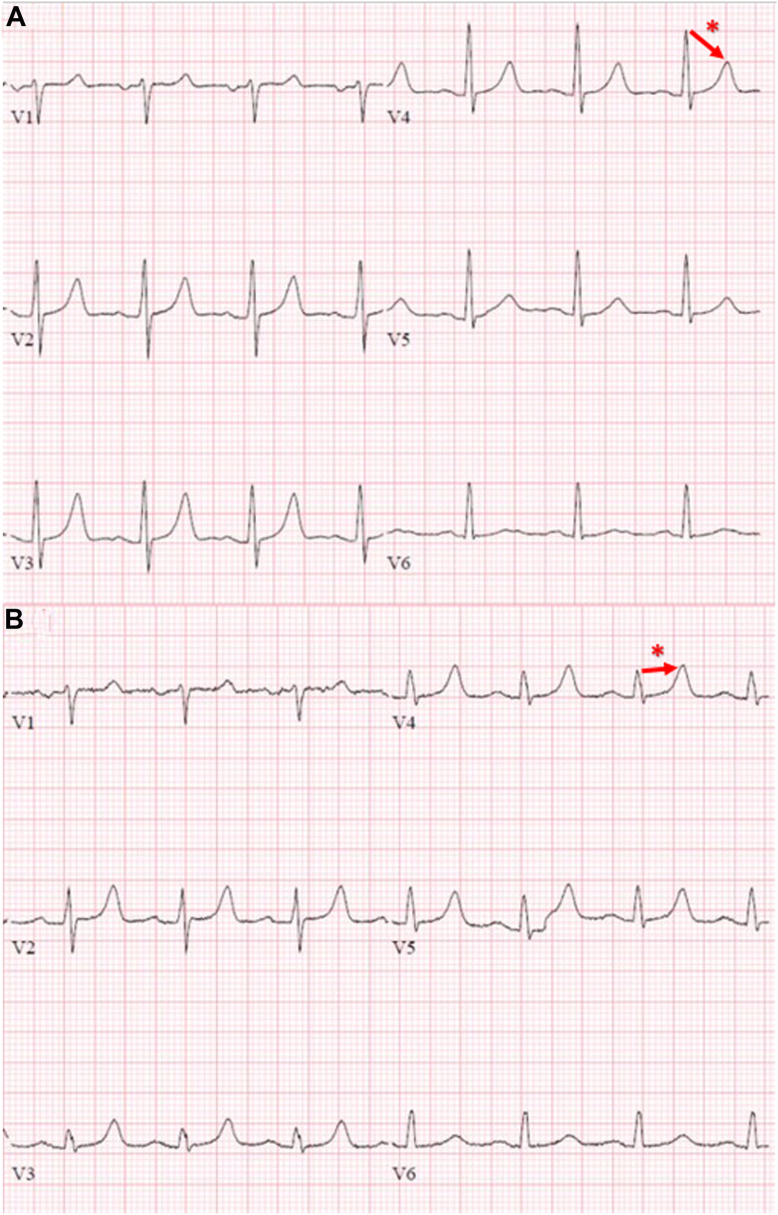
**a:** Preimplantation electrocardiogram (ECG). **b:** Postimplantation ECG demonstrating a change in the QRS/T-wave ratio post defibrillation, visible in the precordial leads. The change in the mean QRS to the mean T-wave ratio through V_1_ to V_6_ leads was 8.3/4.2 (2) before implant vs 4.3/3.8 (1.1) after implant. The V_4_ lead represents the changes in the surface QRS/T ratio. The symbol ∗ illustrates changes in the surface QRS/T ratio in the V_4_ lead, which was 11/5 (2.2) before implant vs 4/5 (0.8) after implant.

A 58-year-old male patient with a past medical history of coronary artery disease with multiple percutaneous coronary interventions; ischemic cardiomyopathy with chronic systolic heart failure, having an ejection fraction of 20%–25% without any improvements despite 3 months of goal-directed medical therapy; class 2 NYHA functional status; and a QRS duration of <90 ms presented for elective S-ICD implantation for primary prevention of sudden cardiac death. The patient was screened exclusively by AST before the implant, passing only the secondary vector in the 2 recommended positions: supine and sitting. The S-ICD was implanted under general anesthesia without significant complications with a successful defibrillation threshold test (DFT) at the end of the case by a single 65-joule shock that demonstrated a shock impedance of 64 ohms. The S-ICD was interrogated in the recovery area, and it was noted upon automation that the secondary vector failed in supine and sitting postures, rendering lack of successful configuration options (Figure 1). The secondary vector failure of the S-ICD raised 2 potential concerns: oversensing of the T wave, leading to inappropriate shocks; and, more importantly, undersensing of ventricular arrhythmia, leading to death. Postprocedure chest radiography showed an unchanged and properly positioned S-ICD generator and thoracic coil (Figure 2). However, 12-lead ECG demonstrated a significant decrease in the QRS-to-T wave ratio postdefibrillation, a significant decrease in the R-wave amplitude, and a slight increase in the T-wave amplitude (see Figure 3). After discussion, we decided to keep the patient under observation in the hospital overnight and reevaluate the following morning. The S-ICD was interrogated the next morning, which subsequently demonstrated appropriate secondary vector configuration screening in the 2 tested positions. Given the inconsistency of the secondary vector to “screen in,” further discussion with the patient was undertaken; however, the patient opted not to undergo implantation of a transvenous device. Repeated DFT was also considered but was not offered, because the second vector AST was appropriate the next day, with the understanding that the patient will follow up closely. The position was also confirmed by fluoroscopy, which again showed the lead/can in the proper positions. The device surgical site was clean, without significant swelling, tenderness to palpation, bleeding, or drainage, and the patient was discharged home without further complications. Repeat testing during follow-up demonstrated passing of the secondary vector in all positions. The patient remains on remote monitoring, with more frequent transmissions scheduled, and has not received any high-voltage therapy on follow-up visits, more than 6 months after this event.

## Discussion

Since its first approval for prevention of sudden cardiac death in 2012, the S-ICD has evolved from first generation to third generation with continuous improvements in size and longevity, automated screening tool with SMART Pass filter, atrial fibrillation monitor, and remote patient management system. In order to ensure the best possible outcomes for S-ICD implantation, preimplantation ECG screening is crucial to predict proper sensing and function postimplantation. The screening process evaluates the ratio of QRS amplitude to T-wave amplitude, and QRST complex in size and duration, and does so both in supine and sitting positions.^5^ Even with this rigorous process, greater than 90% of patients can pass this screening, with the remaining candidates failing preimplant screening largely owing to inadequate QRS sensing or T-wave oversensing.^6^ Once the screening process is complete and passed, during the implantation process the vector selection process uses the Vector Select algorithm, which chooses the vector with the most favorable sensing qualities from the 3 possible sensing configurations. The Vector Select algorithm uses a built-in 0.2–40 Hz bandpass filter for displaying and printing ECG, and adds an S-ICD bandpass filter (3–40 Hz) when computing scores.

There have been many strategies proposed and reported to improve the S-ICD sensing process. Boston Scientific Corporation developed a novel sensing methodology, called the SMART Pass (SP), to overcome cardiac oversensing, which is the main reason for inappropriate shocks from the S-ICD. The SP filter with corner frequency between 8 and 9 Hz and a roll-off rate of 20 dB/decade reduces the amplitude of lower-frequency signals such as T waves, by applying an additional high-pass filter, which lets higher frequencies such as R waves, ventricular tachycardia, and ventricular fibrillation amplitudes to “pass” through. Thus, the practical application of the SP filter is to reduce the amplitude of T waves while QRS waves are being accounted for. The decrease in the QRS/T ratio observed in our study can therefore reduce the accuracy of the SP filter. Theuns and colleagues^7^ evaluated the effect of SP on shocks in ambulatory patients with S-ICD in a prospective blinded cohort study, where they concluded that enabling the SP filter reduces inappropriate shocks by the S-ICD significantly, without a negative effect on appropriate shocks.

Possible mechanisms for vector configuration screening failure and issues after screening include vector change, generator and/or lead disposition, and stability of sensed QRS complexes. Some reports evaluated suitable vector changes and the mechanisms behind them. One such mechanism lies in the change in sensing vector owing to shifts in device position.^8^ This may occur as a result of change in patient position or significant weight change, or during exercise, and may be avoided with reassessment of the automatic setup, such as in significant body habitus changes, assessment in different patient positions, etc.^8^^,^^9^ Another mechanism for vector change may be from changes in myocardial vascularization, as tissue ischemia and different forms of cardiomyopathy may cause dynamic changes in the sensed vectors and/or QRST complexes of patients.^10^

There are also cases where the S-ICD generators and/or leads must be repositioned or stabilized to improve S-ICD implantation, and DFT success^11^ and prevent repetitive inappropriate shocks.^12^ Xiang and colleagues^11^ reported a case of a morbidly obese patient with a migrated S-ICD generator, which was later reanchored with a novel strategy that resulted in a change in the QRST sensing vector, improving the S-ICD implantation and DFT success. Sasaki and colleagues^12^ reported a case of repetitive inappropriate shocks owing to myopotential oversensing, which resolved after lead repositioning.

The screening process is not foolproof, and those deemed eligible might still have failures to sense owing to a change in the initially evaluated eligible vector. The study by Bettin and colleagues^8^ emphasizes the value of having at least 2 acceptable vectors at the time of screening to be able to change vectors manually in follow-up. As mentioned previously, only 1 suitable vector is enough to pass the screening, but there have been suggestions of using a 2/3 leads criterion instead of a “1/3 leads” criterion^6^ with the inclusion of an exercise posture.^13^ Perhaps consideration of a transvenous ICD may be warranted in the setting of a sole vector passing the S-ICD screening.

There is no established evidence-based guideline to manage S-ICD screening failure after its implantation. Firstly, we made sure that the generator and lead positions were intact by using chest radiography. Secondly, we checked the device surgical site for implications of infection, hematoma, and air entrapment; and it was clean without significant swelling, tenderness to palpation, bleeding, or drainage. There were no significant electrolyte abnormalities. The device was interrogated again in supine and sitting positions, which demonstrated proper sensing and function, and now passing the secondary vector. In our case, we suspect the reason of transient vector configuration screening failure postimplantation is mostly related to the significant decrease of QRS amplitude and, to a lesser degree, to increase in the T-wave amplitude, resulting finally in the alteration of the high-pass filter and AST, as described above. Such QRS-wave amplitude decrease and/or T-wave amplitude increase findings have been reported in literature,^14^^,^^15^ but the mechanisms are not fully understood. Hypotheses for these changes include ischemia, myocardial stunning, myocardial edema, and electrolyte changes.^15^ Overall, it is imperative to make sure that the sensing of S-ICD is intact post DFT testing, prior to discharge. This is to allow for the resolution of acute repolarization changes, even when the patient passed the screening in the first place.

## Conclusion

Data outcomes of change or loss in vector screening post S-ICD therapy are scarce. The optimal number of passed screening vectors must be taken into account before and after S-ICD implant, especially in patients who only “screen in” with 1 vector configuration. Our case demonstrates the variability of vector configuration screening with respect to post defibrillation therapy and changes in the surface QRS/T ratio. Fortunately, our patient regained adequate sensing for future detection of ventricular arrhythmia. Future studies should explore preventative and alternative care pathways, should patients only “screen in” successfully to 1 vector configuration, especially in the event that the patient requires more than 1 appropriate therapy from the S-ICD.
